# Tolerance threshold of a pelagic species in China to total dissolved gas supersaturation: from the perspective of survival characteristics and swimming ability

**DOI:** 10.1093/conphys/coae023

**Published:** 2024-05-16

**Authors:** Hongtao Wang, Yuanming Wang, Kefeng Li, Ruifeng Liang, Weiyang Zhao

**Affiliations:** State Key Laboratory of Hydraulics and Mountain River Engineering, Sichuan University, Chengdu 610065, China; State Key Laboratory of Hydraulics and Mountain River Engineering, Sichuan University, Chengdu 610065, China; State Key Laboratory of Hydraulics and Mountain River Engineering, Sichuan University, Chengdu 610065, China; State Key Laboratory of Hydraulics and Mountain River Engineering, Sichuan University, Chengdu 610065, China; State Key Laboratory of Hydraulics and Mountain River Engineering, Sichuan University, Chengdu 610065, China; Institute for Disaster Management and Reconstruction, Sichuan University, Chengdu 610207, China

**Keywords:** Critical swimming ability, gas bubble trauma, loss of equilibrium, mortality, total dissolved gas supersaturation, Yangtze River

## Abstract

Total dissolved gas (TDG) supersaturation downstream of dams can occur in the Yangtze River basin and is known to cause stress and even death in fish. Consequently, it is important to establish tolerance thresholds of endemic fish to protect local aquatic resources. We conducted experiments to assess survival characteristics and swimming ability of bighead carp, an important commercial fish dwelling in the Yangtze River, to evaluate its tolerance threshold to TDG supersaturation. The typical external symptoms of gas bubble trauma (GBT) were observed and the time when the fish lost equilibrium and died were recorded. The results showed that the mortality occurred when TDG level exceeded 125%, with obvious symptoms such as exophthalmos and bubbles on the head. The interval between loss of equilibrium and mortality decreased with an increase in TDG level. Neither exposure time nor TDG level significantly affected the critical swimming speed (U_crit_) of fish exposed to non-lethal exposure (110%, 120% and 125% TDG) over a 7 day period. Significant reductions in U_crit_ were found under 130% and 135% TDG conditions when the exposure lasted 52.0 h and 42.9 h, respectively. The U_crit_ also significantly decreased after exposure of 1.6 h under 140% TDG condition. Moreover, after exposure to 140% TDG for 39.2 h, 135% TDG for 56.5 h and 130% TDG for 95.9 h, bighead carp were transferred into air saturated water to recover for 24 h or 48 h; however, swimming performance remained impaired. The results of this study indicate that 125% TDG was the highest TDG level where limited mortality was observed and the swimming ability was not impaired, showing that 125% TDG can be set as the tolerance threshold of this species to guide the operation of dams in the Yangtze River Basin.

## Teaser

The experiments were conducted to evaluate the tolerance threshold to total dissolved gas supersaturation of bighead carp from the perspective of survival characteristics and swimming ability, which provides a guide for the operation of dams in the Yangtze River Basin.

## Introduction

Total dissolved gas (TDG) supersaturation occurs when the pressure of dissolved atmospheric gases exceeds the local barometric pressure, which can be caused by natural and anthropogenic processes ([Bibr ref10][Bibr ref10], 1984). TDG supersaturation generated by dam spilling is known to have lethal effects on aquatic organisms and has become an issue of urgent concern to all sectors of society (Witt *et al.,* 2017; [Bibr ref23][Bibr ref23], 2019; [Bibr ref67][Bibr ref67], 2022). During the dam discharge period, water pours down into the stilling basin with a sharp increase in peripheral pressures and air entrainments (Li *et al.,* 2022). As the hydrostatic pressure increases, more entrained gases are forced to dissolve at depth. When that water returns to the surface, the dissolved gases exceed their solubility of water at surface pressures and this leads to TDG supersaturation ([Bibr ref19][Bibr ref19], 1997). The TDG supersaturation level is normally less than 105% in the Yangtze River basin without water discharge from dams. However, high levels of TDG supersaturation ranging from 114.9% to 144.2% could occur downstream of dams following water discharge ([Bibr ref43][Bibr ref43], 2011). The TDG supersaturation dissipates slowly and persists a long distance for several days (Ma *et al.,* 2018; [Bibr ref25][Bibr ref25], 2019). For instance, the TDG level was still 117% at 600 km downstream the Three Gorges Dam while the maximum TDG level downstream the dam even reached 143.0% (Qu *et al.,* 2011).

The survival of fish under the stress of TDG supersaturation is characterized by the symptom of gas bubble trauma (GBT) and survival time of fish (Nebeker and Brett, 1976; Gale *et al.,* 2004; [Bibr ref8][Bibr ref8], 2019). The survival of fish has become a particular concern for hydropower station managers because fish death incidents can directly trigger public attention (CCTV,^2014^; Xue *et al.,* 2019). The supersaturated TDG generated during high dam spills may cause GBT for fish and ultimately result in mortality ([Bibr ref30][Bibr ref30], 2009). The gases enter the body when fish breathe and participate in blood circulation, finally causing embolisms in organs ([Bibr ref49][Bibr ref49], 2013; Pleizier *et al.,* 2019; Ji *et al.,* 2021). The external symptoms of GBT manifest as gas bubbles in the lateral line, behind the eyes, between the fin rays, under the skin, in the buccal cavity area and in the blood ([Bibr ref61][Bibr ref61][Bibr ref61][Bibr ref61]). The mortality may result from the effects caused by tissue necrosis and lower immunity ([Bibr ref11][Bibr ref11], 1998; Schisler *et al.,* 2000; Geist *et al.,* 2013). Some endemic species such as rock carp (*Procypris rabaudi*), Prenant’s schizothoracin (*Schizothorax prenanti*) and elongate loach (*Leptobotia elongata*) in the Yangtze River Basin died with apparent symptoms of hyperemia and gas bubbles in the fins under the exposure of over 115% TDG supersaturation during water discharge from dams (Liu *et al.,* 2021; Yuan *et al.,* 2021). The flood discharge of Xiluodu dam located in the Yangtze River caused a total of 40 tons of fish killed due to TDG supersaturation in 2014, which aroused wide social concerns (CCTV,^2014^).

Fish rely on their fundamental property of swimming ability to reproduce, avoid danger and communicate ([Bibr ref44][Bibr ref44], 2000; Plaut, 2001; [Bibr ref45][Bibr ref45], 2022). Once suffering from GBT, bubbles accumulating in the fins affect the balance and movement of fish, and bubbles released from the gill filament block blood vessels and compromise oxygen delivery ([Bibr ref61][Bibr ref61][Bibr ref61][Bibr ref61]). Gas embolism and congestion in the fish’s muscles also reduce their athletic ability (Weitkamp,^2008^). As a result, the fish lose the ability of movement and face the increased risk of predation, which is also a source of indirect mortality for fish ([Bibr ref33][Bibr ref33][Bibr ref33][Bibr ref33]). It is demonstrated that the swimming ability of fish deteriorated by TDG supersaturation could recover after returning to freshwater for a certain time (Schiewe,^1974^; Dawley and Ebel, 1975). The swimming ability of Prenant’s schizothoracin exposed to 130% TDG supersaturation for 2 h returned to normal levels after 2 days of freshwater recovery ([Bibr ref56][Bibr ref56], 2018).

At present, several studies on the tolerance characteristics and swimming ability of fish under TDG supersaturation have been carried out in the Columbia River system (Schiewe, 1974; Dawley and Ebel, 1975; Kovac *et al.,* 2022) and Yangtze River basin ([Bibr ref54][Bibr ref54], 2017; Yuan *et al.,* 2021; Cao *et al.,* 2022; [Bibr ref21][Bibr ref21], 2022). Most of these studies show that the swimming ability of fish was reduced after being stressed by supersaturated TDG water (Schiewe, 1974; Dawley and Ebel, 1975; Ji *et al.,* 2022). Some studies focused on the benthic rare fishes in the Yangtze River, such as rock carp, elongate loach and Prenant’s schizothoracin, but there is still a lack of knowledge on the survival characteristics and swimming ability of pelagic fish under TDG supersaturation ([Bibr ref55][Bibr ref55], 2015a; Wang *et al.,* 2017; Yuan *et al.,* 2021). Gas solubility increases with the increasing water depth, and thus TDG supersaturation decreases by 9.7% for every 1 m increase in water depth ([Bibr ref62][Bibr ref62], 2003; Wang *et al.,* 2020; Pleizier *et al.,* 2021). This also suggests that the pelagic fishes inhabiting in the Yangtze River are more susceptible to TDG supersaturation and face a greater threat of TDG supersaturation than benthic species considering the existence of compensation depth in the natural river (Ji *et al.,* 2021). Therefore, there is an urgent need to address the issue of mortality and swimming ability for pelagic fish stressed by TDG supersaturation.

Bighead carp (*Hypophthalmichthys nobilis*) is widely distributed as a common pelagic fish in the Yangtze River, which is recognized as one of the most commercially important freshwater fish species and has been introduced worldwide ([Bibr ref51][Bibr ref51], 2021). The threshold tolerance of bighead carp to TDG supersaturation is not known and was explored in this paper by investigating survival characteristics and swimming ability in order to reflect the response pattern of bighead carp to TDG supersaturation. On the basis of the previous studies, we proposed hypotheses: (i) there is a threshold of TDG supersaturation for bighead carp, beyond which would result in mortality with obvious GBT symptoms; (ii) the swimming performance would be affected when TDG supersaturation level exceeds a threshold; and (iii) the swimming performance of bighead carp could recover significantly after returning to freshwater for 24 or 48 h. It is expected that our study will provide a reference for protecting pelagic fishes under the stress of supersaturated TDG and formulate water environment protection standards.

## Materials and Methods

One thousand experimental bighead carps with a body length of 55.0 ± 0.4 mm and mass of 3.56 ± 0.08 g were obtained from the Fisheries Institute of the Sichuan Academy of Agricultural Sciences in Chengdu, China. The animal study proposal was approved by the Ethics Committee for Animal Experiments of Sichuan University (2019062101). All experimental procedures were performed in accordance with the regulations for the Administration of Affairs Concerning Experimental Animals approved by the State Council of the People’s Republic of China. The fish were temporarily acclimated in the laboratory for more than 48 h before the experiment without feeding, and they also remained starved throughout the experiment. The static water used for acclimating fish was replaced once every 3 days. The measured dissolved oxygen (DO) concentration was 8.8 ± 0.6 mg/L and the water temperature was 16.8 ± 1.4°C during the acclimation.

Two experiments from the perspective of survival and swimming ability were conducted in this study to identify the tolerance threshold of bighead carp to TDG supersaturation. According to previous articles, special attention should be paid to the symptoms of GBT and the lethal time of fish when studying the stress of TDG supersaturation on fish ([Bibr ref12][Bibr ref12][Bibr ref12][Bibr ref12]; [Bibr ref61][Bibr ref61][Bibr ref61][Bibr ref61]; Colt *et al.,* 1984; Li *et al*., 2022). We carried out the survival experiment by recording external symptoms of GBT, time to loss of equilibrium (LOE) and time to mortality. The swimming ability of bighead carp was evaluated by testing the critical swimming speed (U_crit_) under different TDG supersaturation exposure. The U_crit_ was also tested after 24 or 48 h of recovery from 130%, 135% and 140% TDG supersaturation exposure. The tolerance threshold of bighead carp was finally identified based on the results of survival and swimming ability experiments.

### Survival experiment

Seven exposure groups were set under the nominal TDG level of 100% (control group), 110%, 120%, 125%, 130%, 135% and 140%. For each TDG condition, 31 or 40 fish were introduced to the tank with length × width × height of 70 × 40 × 35 cm (see detailed information in Supplementary Table S8, see online supplementary material) and the replication was considered at the individual level. The water temperature was 16.3 ± 0.7°C during the survival experiment. 

TDG supersaturated water was generated by simultaneously introducing saturated water and air into the pressure vessel through a pump, which was described in more detail by Wang *et al.* (2015b). The flow rates of supersaturated and equilibrium (100% TDG) water were adjusted by opening and closing valves to create the desired TDG levels at each tank. It must be noted that we cannot get a constant TDG level and each condition had a fluctuation in TDG level within 2.1% and a fluctuation in DO within 4.6% (Table 1). The detailed parameters for each TDG and DO condition can be seen in Supplementary Tables S1–S7 (see online supplementary material). A TDG measuring instrument (PT4Tracker; Point Four Systems, Coquitlam, British Columbia, Canada) was used to track the level of TDG supersaturation. A water quality measuring instrument (YSI PRO20i, YSI Inc., Yellow Springs, OH, USA) was used to record the dissolved oxygen and water temperature.

**Table 1:** The summary of TDG and DO values for each treatment condition in the survival and swimming ability experiment

	TDG treatment	TDG (%)	DO (%)
	100%	101.5 ± 0.4	94.0 ± 1.7
	110%	110.1 ± 2.1	107.5 ± 2.2
	120%	119.2 ± 0.9	115.8 ± 3.7
Survival experiment	125%	124.4 ± 0.9	119.2 ± 4.6
	130%	129.4 ± 1.1	124.1 ± 3.3
	135%	135.2 ± 1.1	129.3 ± 4.6
	140%	139.7 ± 0.9	133.3 ± 4.4
	100%	101.6 ± 0.9	94.8 ± 2.5
	110%	110.9 ± 2.0	106.7 ± 2.7
	120%	119.9 ± 0.8	115.0 ± 3.5
Swimming ability experiment	125%	124.3 ± 0.9	118.7 ± 3.8
	130%	130.9 ± 1.2	122.8 ± 2.8
	135%	134.8 ± 0.4	129.3 ± 3.7
	140%	140.3 ± 0.5	133.9 ± 2.9
Recovery experiment	100%	101.5 ± 0.5	94.4 ± 1.5

The values of TDG and DO are shown as mean ± SD.

The experimental fish were directly introduced into each tank at the same time in groups after the desired TDG levels were reached. The detailed information on the group size, fish body length and mass in each tank is shown in Supplementary Table S8 (see online supplementary material). The behavior of the fish was constantly monitored all the time throughout the experiment, and the time to LOE and mortality for each fish were recorded. Fish generally lose equilibrium before reaching mortality. LOE for bighead carp in this study was defined as the failure to maintain dorsal-ventral orientation for more than a half minute. Some fish were surrounded by bubbles and white foam when they lost equilibrium. The dead fish in the TDG supersaturated water generally float on the water surface or move with the current. Bighead carp were determined to have reached mortality when they had no reaction after being prodded four consecutive times. Dead fish were removed from TDG supersaturated water immediately and then checked the GBT signs, including hyperemia and bubbles in the fins. The GBT symptoms of dead fish were observed under a digital microscope. The mass and length of each dead fish were also recorded at the sametime.

### Swimming ability experiment

The time of mortality could provide an important reference to the experimental setup of more complex physiological traits ([Bibr ref56][Bibr ref56], 2018; Ji *et al.,* 2022). Therefore, we set the timepoints in the swimming ability experiment according to the time when the first mortality occurred and the time when a certain percentage of mortality was reached.

Obvious mortality occurred when TDG supersaturation was over 125%, while fish survived well when TDG supersaturation was lower than 125%. We therefore set 110%, 120% and 125% TDG supersaturation as the non-lethal conditions (where fish did not suffer mortality and survived well). Sixty fish for each non-lethal condition were distributed in one tank for 7 days of TDG supersaturation exposure. The fish exposed in 100% TDG level were tested as the control group. The fish in groups of seven were randomly selected and transferred into the swimming tunnel respirometer for U_crit_ test every 24 h during the 7 days of exposure. Fish were treated as individual replicates and each experimental fish was used only once. Swimming ability was tested at 9:00 a.m., 12:00 p.m. and 3:00 p.m. for 110%, 120% and 125% TDG supersaturation each day, respectively. The U_crit_ of the bighead carp kept in the equilibrium water was tested for comparison. The water temperature was 16.0 ± 0.7°C during the non-lethal swimming ability experiment. The detailed parameters for each non-lethal condition can be seen in Table 1 and Supplementary Tables S9–S12 (see online supplementary material). The details of experimental equipment are described below.

The lethal conditions were set at 130%, 135% and 140% TDG supersaturation (where acute mortality of fish occurred with exposure time) and over 140 fish were exposed to each lethal TDG condition. According to the result of the survival experiment, the first mortality of fish under 140%, 135% and 130% TDG condition were found at 6.3 h, 29.3 h and 23.3 h of exposure, respectively. 30% mortality was reached at 56.5 h and 95.9 h of exposure for 135% and 130% TDG conditions, while 20% mortality occurred at 17.8 h of exposure for 140% TDG conditions. Therefore, the U_crit_ under 140% TDG condition were tested at 0.8 h, 1.6 h, 3.2 h, 6.3 h, 10.4 h and 17.8 h of exposure to evaluate the swimming ability of fish. The U_crit_ of fish under 135% TDG condition were tested at 7.4 h, 14.8 h, 29.3 h, 42.9 h, 50.8 h and 56.5 h respectively, while the U_crit_ of fish under 130% TDG condition were tested at 5.8 h, 11.6 h, 23.3 h, 52.0 h, 60.7 h, 72.5 h, 80.8 h and 95.9 h, respectively. The fish exposed in 100% TDG level were regarded as the control group. Seven fish were randomly chosen to test the U_crit_ at each time point and each experimental fish was used only once. The water temperature was 18.6 ± 1.2°C during the lethal swimming ability experiment. The detailed parameters for each lethal condition can be seen in Table 1 and Supplementary Tables S13–S15 (the lethal conditions).

The TDG supersaturation exposure was stopped at 39.2 h for 140% TDG condition, 56.5 h for 135% TDG condition and 95.9 h for 130% TDG condition. All the surviving fish under each lethal condition were then transferred into the equilibrium water for recovery. The U_crit_ of fish after 24 h (for 130%, 135% and 140% TDG conditions) and 48 h (for 135% and 140% TDG conditions) of recovery were tested and each experimental fish was used only once. The U_crit_ of fish in each treatment group was compared with the control group (100% TDG) and the exposure endpoint group, respectively. The detailed parameters for each recovery group are shown in Table 1 and Supplementary Table S16 (see online supplementary material). The information on body length and mass of bighead carp under each TDG condition and recovery group are listed in Supplementary Tables S17–S19 (see online supplementary material).

A swimming tunnel respirometer (Loligo Systems SW10150, Denmark) was used to measure the U_crit_. The volume of the sealing area of the circular experimental tank is 30 L and the swimming chamber has a volume of 9 L (length × width × height: 46 × 14 × 14 cm). The cross-sectional area of 7 fish was < 10% of the swimming chamber in this study so the mutual influence of fish and the fish obstruction of flow could be negligible (Pang *et al.,* 2010). The front rectifier grid is set at the inlet of the swimming chamber, which is a fine honeycomb partition to stabilize the uniform distribution of flow velocity. Two probes are also set at the inlet to record the conditions of water temperature and dissolved oxygen, respectively. A wire mesh is fixed in the downstream of the swimming chamber to prevent fish from escaping. The experimental tank is all made of transparent plexiglass, and therefore the swimming behavior of fish can be clearly observed from the side andtop.

U_crit_ was determined by the increasing velocity method ([Bibr ref20][Bibr ref20], 1997). Fish were put in the swimming chamber under a 5 cm/s flow velocity for 10 minutes to eliminate the effect of the transfer process before the swimming ability test. The flow velocity was then steadily increased at an increment of 10 cm/s every 20 minutes from 10 cm/s until the fish was exhausted and leaned against the downstream wire mesh for more than 2 minutes. The U_crit_ was calculated as follows (Brett, 1964):

$$ {U}_{crit}=v+\left(T/\triangle T\right)\times \triangle v $$

where $ v$ is the secondary maximum velocity when the fish was exhausted, $\triangle v $ is the flow velocity increment (10 cm/s in the present study), $ \triangle T$ is the duration of each flow velocity (20 min in the present study) and $ T$ is the length of time that fish swam at the maximum flow velocity before they were exhausted.

### Statistical analysis

Analyses were performed using data collected in all experiments. The Kaplan–Meier method was used to analyse the survival rate of fish under different TDG conditions. Two-way ANOVA was used to analyse the U_crit_ of experimental fish under different exposure time and TDG supersaturation in non-lethal groups. The effect of lethal TDG exposure on the U_crit_ was tested by one-way ANOVA. Post hoc multiple comparison test (least significant difference test) was used to compare the swimming ability of bighead carp under different groups. The independent samples *t*-test was used to compare the difference in U_crit_ between the control group and each recovery group. Statistical significance was set at *P* < 0.05.

## Results

### Survival characteristics of bighead carp under TDG supersaturation

#### The external symptoms of GBT

**Figure 1:**
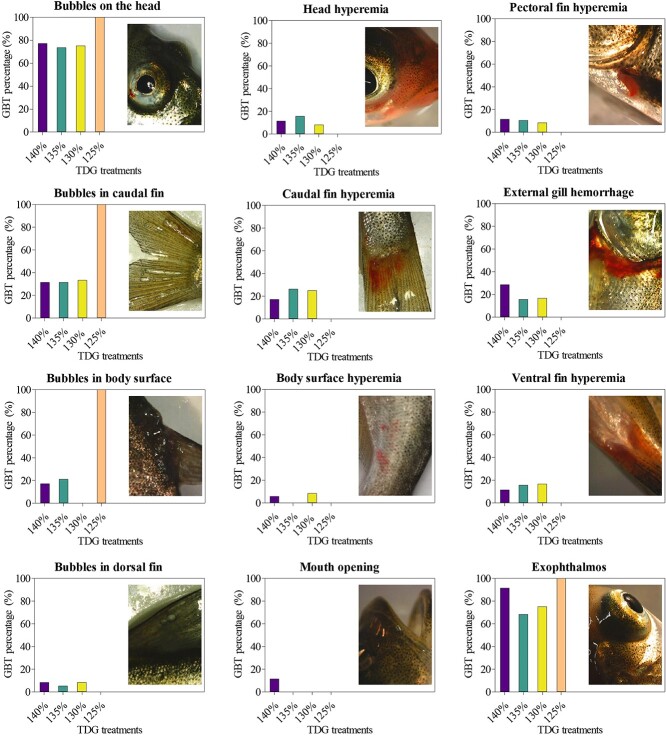
Typical external gas bubble trauma symptoms of bighead carp in TDG supersaturated water. The proportions of different symptoms are indicated as bars and the corresponding image of each symptom is also shown in the figure. External gas bubble trauma symptoms of 100%, 110% and 120% TDG treatments were not shown due to no mortality.

Bighead carp exposed to higher TDG supersaturated water were observed to gradually move to the water surface and finally suffer from mortality with the increase of exposure time. The symptoms of GBT mainly appeared on fish in 130% or higher TDG supersaturated water, while external symptoms were not evaluated under 125% TDG supersaturation due to no mortality (Fig. 1). The proportions of exophthalmos and bubbles in the head of experimental fish were more than 60% above 125% TDG level. The proportions of bubbles in the caudal fin were also relatively higher, which were over 20% in each condition of TDG supersaturation. Other symptoms had relatively lower proportions and even were not found in some TDG conditions.

#### The survival rate of bighead carp

**Figure 2:**
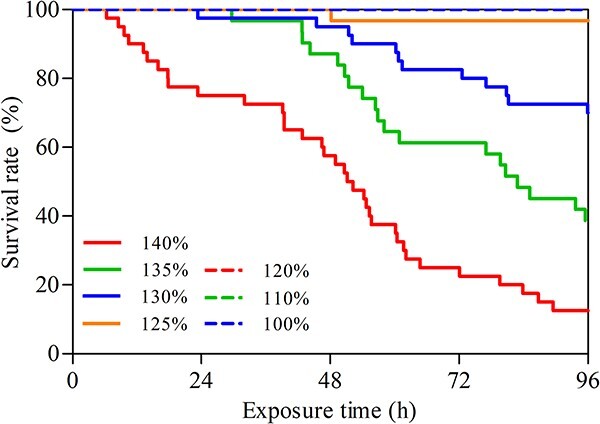
Survival curve of bighead carp under different TDG supersaturations. No mortality was found in 100%, 110% and 120% TDG supersaturated water.

No mortality occurred in 100%, 110% and 120% TDG conditions. Mortality was found in the 125%, 130%, 135% and 140% TDG supersaturated water, and the first death appeared at 48.17, 23.28, 29.63 and 6.33 h of TDG supersaturation exposure, respectively (Fig. 2). The survival rate of bighead carp decreased with increasing TDG supersaturation, and the final survival rate of fish in 130%, 135% and 140% TDG was 70.00%, 38.71% and 12.50%, respectively. Only one fish was dead in the 125% TDG level. As shown in Supplementary Table S20 (see online supplementary material), the survival of bighead carp was significantly different among the conditions where TDG level was equal to and over 125% (*P* < 0.05). No significance in survival rate was found between non-lethal groups (100%, 110% and 120% TDG) and the 125% TDG group (*χ2* = 1.290, *P* = 0.256).

#### Time to loss of equilibrium

**Figure 3:**
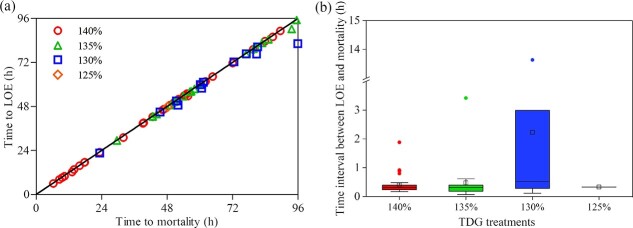
The time to LOE and the time to mortality of each fish under different TDG levels (a) and the time interval between LOE and mortality under each TDG exposure group (b). Each point in (a) refers to an individual fish. The x and y-axis refer to the time when a fish died and reached LOE, respectively. The boxes in (b) illustrate the range of lower and upper quartiles. The whiskers indicate 1.5 interquartile ranges. The medians are shown inside the boxes and the outliers are shown by the plots outside the boxes. 100%, 110% and 120% TDG treatments were not shown due to no mortality.

Bighead carp were found LOE before suffering mortality. As shown in Fig. 3a, the time to LOE and mortality was quite close for fish under each TDG condition. The maximum time interval between LOE and mortality was found in the condition of 130% TDG with a value of 13.63 h. The average time interval was 24.20 min, 28.42 min and 133.75 min, respectively, under the condition of 140%, 135% and 130% TDG (Fig. 3b). Only one bighead carp died in the 125% TDG supersaturated water, and the time interval was 20 min.

### Swimming ability of bighead carp under TDG supersaturation

#### Swimming ability under non-lethal TDG supersaturation

**Figure 4:**
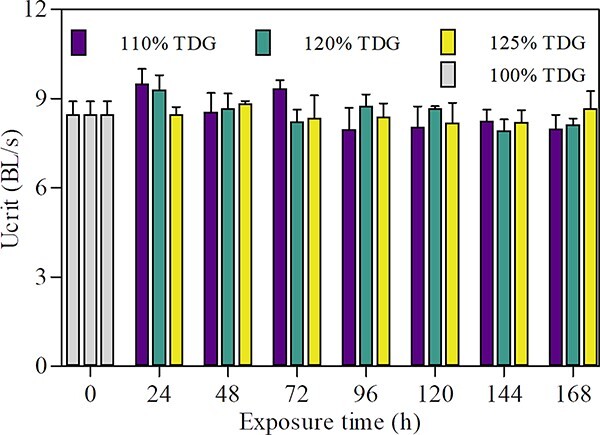
U_crit_ of bighead carp at varying exposure times under non-lethal TDG supersaturation. The values of U_crit_ are expressed as mean ± SE, and no significant difference was found in U_crit_ among exposure time and TDG supersaturation. U_crit_ at 0 h represents the result of control group (fish exposed to 100% TDG level).

As shown in Fig. 4, the U_crit_ of bighead carp in 110%, 120% and 125% supersaturated TDG water was between 6.38–9.57 BL/s, 7.56–8.92 BL/s and 6.30–10.80 BL/s, respectively, under 7-day of non-lethal TDG supersaturation exposure. The U_crit_ were 8.00 BL/s, 8.14 BL/s, 8.68 BL/s after 7 d of exposure, which accounted for 94.4%, 96.0%, 102.3% compared to the control group, respectively. Two-way ANOVA analysis showed that exposure time (*F_(7,14)_* = 1.251, *P* = 0.279), TDG supersaturation (*F_(2,14)_* = 0.055, *P* = 0.946) and their interaction (*F_(14,144)_* = 0.703, *P* = 0.769) did not have a significant effect on fish swimming ability.

#### Swimming ability under lethal TDG supersaturation

**Figure 5:**
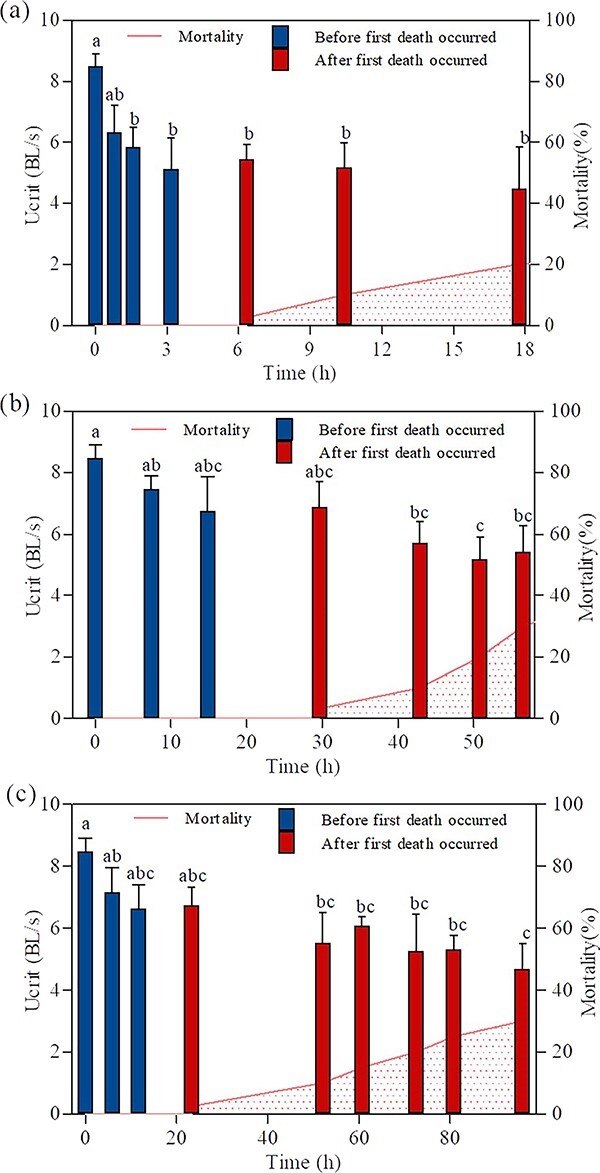
U_crit_ of bighead carp at varying exposure times under 140% (a), 135% (b) and 130% (c) TDG supersaturation. The values of U_crit_ are expressed as mean ± SE. U_crit_ of fish before any deaths are indicated in blue while U_crit_ where some deaths were observed in that treatment are indicated in red. Letters above the bars indicate the results of a post hoc multiple comparison test (least significant difference test); mean values that do not share a common lowercase letter are significantly different (*P* < 0.05). The curves in the figure indicate mortality of fish under 130% (a), 135% (b) and 140% (c) TDG supersaturation. The group tested at 0 h refers to the control group (fish exposed to 100% TDG level).

The swimming ability of bighead carp was significantly affected by the exposure of lethal TDG supersaturation compared with the control group of 8.48 BL/s (one-way ANOVA for 140% TDG: *F_(6, 33)_* = 2.884, *P* = 0.023; for 135% TDG: *F_(6, 37)_* = 2.545, *P* = 0.037; for 130% TDG: *F_(8, 45)_* = 2.895, *P* = 0.011). Significant decrease in U_crit_ for 140% TDG occurred at 1.6 h of exposure with a value of 5.84 BL/s (Fig. 5a). For 135% and 130% TDG, the significant decreases in U_crit_ were observed after exposure of 42.9 h and 52.0 h with values of 5.71 and 5.53 BL/s, respectively (Fig. 5b and c). The U_crit_ of fish at 17.8 h of 140% TDG exposure was 4.48 BL/s, which decreased 47.1% compared with that of the control group. The U_crit_ after exposure of 42.9 h and 52.0 h under 135% and 130% TDG conditions had 36.0% and 44.9% of reduction compared with that of the control group, respectively.

#### Swimming ability after recovery

**Figure 6:**
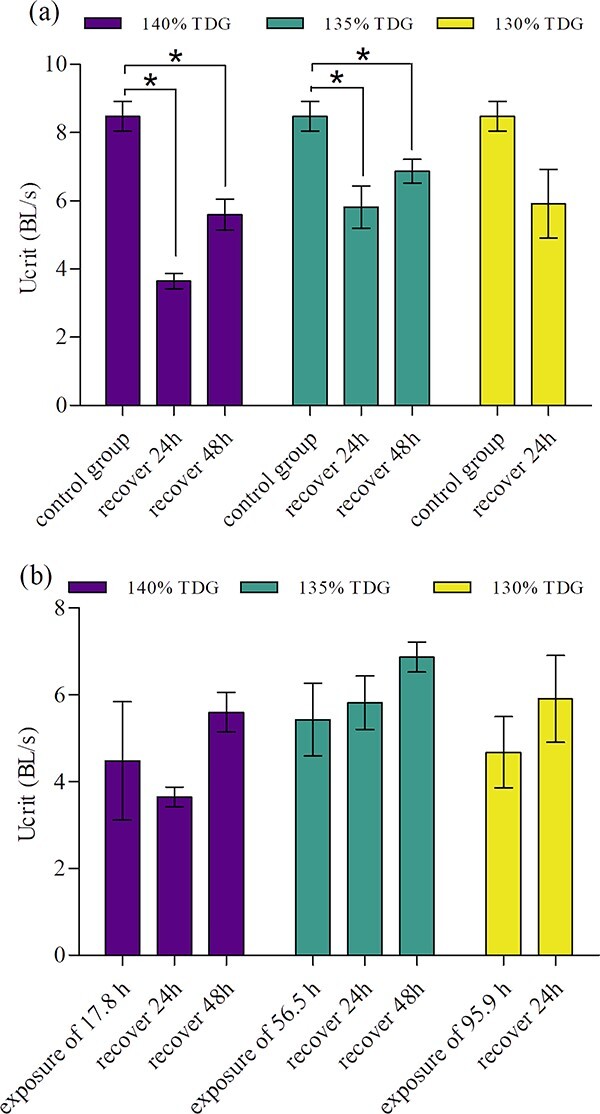
Comparison of U_crit_ of recovery groups with the control group (a) and the TDG exposure groups (b). The values of U_crit_ are expressed as mean ± SE. * shows a significance difference between two groups. The fish exposed to the 100% TDG level were regarded as the control group.

After being transferred to the equilibrium water for 24 h and 48 h of recovery, the U_crit_ of bighead carp suffered 39.2 h of 140% TDG exposure reached 3.65 and 5.60 BL/s, respectively (Fig. 6a). However, 24 h and 48 h of recovery did not let fish reach the normal level compared to that of the control group (independent *t*-test for 24 h of recovery: *F_9_* = 1.370, *P* = 0.000; for 48 h of recovery: *F_8_* = 0.223, *P* = 0.004). The U_crit_ of bighead carp exposed to 135% TDG for 56.5 h reached 5.82 BL/s and 6.87 BL/s respectively after 24 h and 48 h of recovery, but were also significantly lower than that of the control group (independent t-test for 24 h of recovery: *F_12_* = 1.853, *P* = 0.004; for 48 h of recovery: *F_12_* = 0.266, *P* = 0.013). For fish exposed to 130% TDG for 95.9 h, its U_crit_ increased to 5.91 BL/s after 24 h of recovery with a large variation of 2.22–8.68 BL/s, although there was no significant difference with the control groups (independent *t*-test: *F_6.827_* = 5.283, *P* = 0.051). In addition, there was no significant difference in U_crit_ between the recovery group and the exposure endpoint group (Fig. 6b). Generally, the swimming ability of bighead carp acclimated to the equilibrium water after lethal TDG exposure could not recover within just 48 h.

## Discussion

The study explored the response pattern of bighead carp to TDG supersaturation from the perspective of survival characteristics and swimming ability. The results of this study could provide a reference for protecting pelagic fishes under the stress of supersaturated TDG and formulating water environment protection standards. It also could guide the operation of dams in the Yangtze River Basin. Obvious external GBT symptoms and mortality were found when the level of TDG supersaturation was over 125%. The exposure of TDG supersaturation to less than 125% did not significantly change the swimming ability of bighead carp. The swimming ability of bighead carp significantly decreased when exposed to higher than 125% TDG supersaturated water, and could not recover within 24 or 48 h. The results of this study suggested that 125% is the threshold of bighead carp to TDG supersaturation with 7 d of TDG supersaturation exposure.

### Survival characteristics of bighead carp under TDG supersaturation

It has been well established that special attention should be paid to the symptoms of GBT when studying the stress of TDG supersaturation on fish according to previous articles ([Bibr ref12][Bibr ref12][Bibr ref12][Bibr ref12]; [Bibr ref61][Bibr ref61][Bibr ref61][Bibr ref61]; [Bibr ref10][Bibr ref10], 1984; Li *et al*., 2022). Bighead carp exhibited bubble formation under the skin and fins and then gradually lost equilibrium under lethal TDG exposure, which was regarded as an obvious reflection of GBT (Bouck,^1980^; Espmark *et al.,* 2010). Viscous foam secreted by fish can be clearly observed on the water surface during the process of TDG supersaturation exposure, which is treated as a regulatory mechanism for fish to actively adapt to changes in environmental factors. Viscous foam made bighead carp have a certain resistance to TDG supersaturation (Shephard,^1994^). The exophthalmos and bubbles in the head were found to have relatively higher probabilities in the juvenile bighead carp in this study. The precipitation and convergence of a large number of bubbles at the back of the cornea caused the relative displacement of the eyeball and the deformation of the periocular tissue, and finally resulted in exophthalmos (Weitkamp and Katz, 1980; Krise and Smith, 1993). The symptoms of bubbles in fins and gill hemorrhage were also very obvious in juvenile bighead carp. The fins are the main organs that drive fishes to move forward and keep their balance ([Bibr ref16][Bibr ref16][Bibr ref16][Bibr ref16]). The bighead carp were exposed to supersaturated TDG water and their moving resulted in a higher bubble occurrencerate.

The survival of fish has become an unavoidable issue for hydropower station managers due to the high attention of public opinion (CCTV,^2014^; Xue *et al.,* 2019). The previous studies also used lethal time to quantify the impact of supersaturated TDG on fish ([Bibr ref57][Bibr ref57], 2020; Yuan *et al.,* 2020; Ji *et al.,* 2021; [Bibr ref31][Bibr ref31], 2021; [Bibr ref66][Bibr ref66], 2021). Fish mortality occurred in the present study when the TDG level exceeded 125%. Similarly, Ji *et al.* (2021) also found the silver carp (*Hypophthalmichthys molitrix*), belonging to the same genus as bighead carp, did not die below 130% TDG supersaturation for 72 h of exposure. However, some endemic fish in the Yangtze River basin were found to have relatively lower lethal TDG levels compared to these two carps ([Bibr ref57][Bibr ref57], 2020; Liu *et al.,* 2021; Yuan *et al.,* 2021). For example, 50% of juvenile rock carp died after 14 h of 120% TDG exposure (Wang *et al.,* 2020). For Prenant’s Schizothoracin, mortality occurred during 96 h of 120% TDG exposure ([Bibr ref57][Bibr ref57], 2020). Juvenile chinook salmon (*Oncorhynchus tshawytscha*) held in 124% TDG supersaturated water with a depth of 0.6 m suffered 92–100% mortality in 5–7 days (Weitkamp and Katz, 1980). The mountain whitefish (*Prosopium williamsani*) and cutthroat trout (*Salmo clarki*) held in shallow live-cages were dead with signs of GBT within 96 h at TDG supersaturation above 130% ([Bibr ref61][Bibr ref61][Bibr ref61][Bibr ref61]). It is indicated that rock carp, Prenant’s Schizothoracin, chinook salmon, mountain whitefish and cutthroat trout are more susceptible than bighead carp under the exposure of TDG supersaturation.

### Swimming ability of bighead carp under TDG supersaturation

The U_crit_ of the bighead carp under non-lethal TDG exposure (125% or below) kept stable compared with the control groups. The fish still owned normal swimming ability and limited mortality occurred after even 7 d of TDG exposure. It can be inferred that the effect of low TDG exposure on bighead carp was limited and did not significantly affect the foraging or cruising. Similar to the bighead carp, the swimming ability of grass carp and silver carp was also found to not be significantly affected under the non-lethal TDG exposure (Cao *et al.,* 2022; [Bibr ref21][Bibr ref21], 2022). However, the U_crit_ of the Chinese sucker (*Myxocyprinus asiaticus*) and Prenant’s Schizothoracin decreased significantly when exposed to 117% TDG supersaturated water ([Bibr ref56][Bibr ref56], 2018). It can be concluded that bighead carp has certain advantages in resisting TDG supersaturation compared with Chinese sucker and Prenant’s Schizothoracin.

The result of this study shows that there was a significant difference in U_crit_ after 1.6 h of exposure under 140% TDG conditions compared to the control group. Under 135% and 130% TDG conditions, significant differences in U_crit_ were found after exposure of 42.9 h and 52.0 h compared to the control group, respectively. The above result demonstrates that the swimming ability of bighead carp would suffer impairment with obvious GBT symptoms and LOE when the TDG supersaturation was over 125%. It means that behaviors of bighead carp closely related to swimming ability, such as foraging behavior, evasion capacity and propagation, may be impaired in higher TDG supersaturated water due to reduced swimming ability (Webb,^1984^). The result of this study also shows that the swimming ability of bighead carp failed to recover in the equilibrium water after exposure of 39.2 h, 56.5 h and 95.9 h under 140%, 135% and 130% TDG conditions, respectively. However, some studies showed other fishes returned to the equilibrium water appeared to recover from TDG exposure. For instance, external symptoms of GBT were no longer observed on steelhead trout after 15 d of recovery (Dawley and Ebel, 1975). The swimming speeds of Prenant’s Schizothoracin showed significant recovery after 2 d ([Bibr ref56][Bibr ref56], 2018). The U_crit_ of elongate loach recovered to the normal level after experiencing more than 28 h (Yuan *et al.,* 2021). The grass carp (*Ctenopharyngodon idella*) almost completely recovered to the normal state of swimming after returning to the equilibrium water for 2 h ([Bibr ref6][Bibr ref6], 2022). We speculate that bighead carp had been severely impaired under lethal TDG exposure, making them hard to recover in the equilibrium water.

### Tolerance threshold of bighead carp to TDG supersaturation

Fish can resist the pressure of supersaturated TDG through self-regulation within a certain TDG threshold range. Higher TDG supersaturation than this threshold would result in fish suffering behavior damage or even mortality ([Bibr ref61][Bibr ref61][Bibr ref61][Bibr ref61]; [Bibr ref7][Bibr ref7], 2016; Pleizier *et al.,* 2020; Algera *et al.,* 2022). According to the tolerance of salmonids to supersaturated TDG, some states in the northwest of the United States ever took 110% as the limit of TDG supersaturation in the Columbia River basin for management (USEPA,^1976^). In recent years, Washington and Oregon have further improved the limit of TDG supersaturation to 120% in the Columbia River system and allowed TDG supersaturation to reach 125% in some reaches ([Bibr ref63][Bibr ref63], 2017). The Canadian guidelines on TDG supersaturation defined 110% TDG as the threshold in rivers (Canadian Council of Ministers of the Environment, 1999). However, gas saturation guidelines for natural water bodies are non-existent outside of North America, even TDG supersaturation is commonly found in dam-regulated rivers around the world (Pulg *et al.,* 2016; Xue *et al.,* 2019; [Bibr ref50][Bibr ref50][Bibr ref50]; [Bibr ref65][Bibr ref65], 2020; [Bibr ref24][Bibr ref24], 2020). Due to the construction of high dams in the Yangtze River basin, endemic fishes are also severely stressed by TDG supersaturation, and corresponding regulations should be formulated for the tolerance threshold of endemic fishes to TDG supersaturation. At the same time, some measures can be taken to reduce the TDG supersaturation level. Flow deflectors installed on the spillway face are considered as an efficient means to mitigate TDG supersaturation, which may change the plunging flow into a skimming flow and prevent spillway flow from plunging deep into the stilling basin by forcing the spillway jet horizontally ([Bibr ref35][Bibr ref35], 2000; Li *et al.,* 2022). Some operational solutions were also presented such as a spill flow concentration method and a ski-jump energy dissipation method ([Bibr ref41][Bibr ref41]; Feng *et al.,* 2018). In addition, interval and flood pulse discharge patterns could cause a lower TDG level downstream effectively (Feng *et al.,* 2014; Wan *et al.,* 2021).

Considering that the survival characteristics and swimming ability of bighead carp were significantly deteriorated when TDG supersaturation was over 125% in this study, we suggested that 125% can be taken as the tolerance threshold of the bighead carp to TDG supersaturation. It is worth mentioning that the current recommendation is based on 7 days of TDG supersaturation exposure. Larger effects would have been observed at lower TDG levels if the exposure time was much longer and that is what would occur in the wild (Ji *et al.,* 2019; Yuan *et al.,* 2021). Besides this pelagic species, tolerance thresholds from only two fishes (Chinese sucker and silver carp) were obtained in the Yangtze River basin ([Bibr ref7][Bibr ref7], 2016; Ji *et al.,* 2021). Formulating corresponding standards requires more comprehensive studies on fish tolerance to TDG supersaturation.

## Conclusions

This study investigated the tolerance threshold of bighead carp to TDG supersaturation from two aspects, namely survival characteristics and swimming ability. The following conclusions can be drawn through this study:

Mortality of bighead carp occurred within 96 h when TDG supersaturation was equal to or higher than 125%. The survival rate of bighead carp decreased with increasing TDG supersaturation, and the final survival rate of fish in 125%, 130%, 135% and 140% TDG was 96.78, 70.00, 38.71 and 12.50%, respectively. Bighead carp that died exhibited obvious symptoms of GBT and more than 60% fish above 125% TDG supersaturation had exophthalmos and bubbles on thehead.

The U_crit_ of bighead carp under 7 days of 110%, 120% and 125% TDG supersaturation exposure was 8.53 ± 0.19 BL/s, 8.53 ± 0.14 BL/s and 8.46 ± 0.16 BL/s, respectively. The U_crit_ under non-lethal TDG exposure showed no significant difference compared with the control group but was significantly affected by the exposure of lethal TDG supersaturation. The significant decrease in U_crit_ for 140% TDG occurred at 1.6 h while significant decreases were observed after exposure of 42.9 h and 52.0 h for 135% and 130% TDG conditions. Bighead carp under lethal TDG exposure could not recover to normal levels after 24 or 48 h of recovery in the equilibrium water.

Considering that the survival characteristics and swimming ability of bighead carp were significantly deteriorated when TDG supersaturation was over 125%, we suggested that 125% can be taken as the tolerance threshold of the bighead carp to TDG supersaturation.

## Supplementary Material

Web_Material_coae023

## Data Availability

Data will be made available on request.
