# Supplementary material for: Tolerance threshold of a pelagic species in China to total dissolved gas supersaturation: from the perspective of survival characteristics and swimming ability
Source: Conserv Physiol. 2024 May 16;12(1):coae023. doi: 10.1093/conphys/coae023 (PMC11099944; doi:10.1093/conphys/coae023)
Supplement: Web_Material_coae023 [file web_material_coae023.pdf]

Supporting information

Tolerance threshold of a pelagic species in China to total dissolved gas supersaturation: from the perspective of survival characteristics and swimming ability

Hongtao Wang[1], Yuanming Wang[1,*], Kefeng Li[1], Ruifeng Liang[1], Weiyang Zhao[1,2]

[1] State Key Laboratory of Hydraulics and Mountain River Engineering, Sichuan University, Chengdu 610065, China

[2] Institute for Disaster Management and Reconstruction, Sichuan University, Chengdu 610207, China

* Corresponding author: State Key Laboratory of Hydraulics and Mountain River Engineering, Sichuan University, Chengdu 610065, China. Email: wangyuanming1991@126.com

Contents of this file

Table S1 to S20

Table S1: The monitoring parameters for 100% TDG supersaturation group in survival experiment. All the data was recorded at least once per day. The mean TDG saturation was 101.5 ± 0.4%. The mean DO level was 94.0 ± 1.7%. The dashes mean data was not recorded.

Exposure time (h)	Temperature (℃)	TDG (%)	DO (%)	TDG (mmHg)	DO (mg/L)	BP (mmHg)
0.00	14.6	101	95.6	723	9.73	716
11.70	14.8	-	93.6	-	9.47	-
24.00	15.1	101	94.4	721	9.5	715
34.93	16	102	93.9	720	9.49	709
36.50	-	102	-	722	-	710
47.62	17.4	102	99	728	9.73	711
53.32	17.5	102	93.4	720	9.13	709
62.50	16.9	101	93.7	720	9.28	710
70.88	16.8	102	94.3	721	9.33	710
75.13	16.5	101	92.6	719	9.20	708
76.17	16.4	102	93	719	9.22	707
77.32	16.5	102	93	719	9.20	708
86.02	16.2	101	92.4	718	9.21	710
96.00	17.5	101	93.2	719	9.07	710

Table S2: The monitoring parameters for 110% TDG supersaturation group in survival experiment. All the data was recorded at least once per day. The mean TDG saturation was 110.1 ± 2.1%. The mean DO level was 107.5 ± 2.2%. The dashes mean data was not recorded.

Exposure time (h)	Temperature (℃)	TDG (%)	DO (%)	TDG (mmHg)	DO (mg/L)	BP (mmHg)
0.00	14.7	113	106.3	815	10.8	719
11.70	15.3	-	104.3	-	10.44	-
24.00	15.5	115	104.8	820	10.47	715
34.93	15.9	108	105.5	766	10.45	709
36.50	-	109	-	778	-	710
47.62	15.9	110	109.9	780	10.88	711
53.32	16.3	108	104.8	766	10.28	709
62.50	16.3	108	105.6	769	10.35	710
70.88	16.4	109	109.2	775	10.69	710
75.13	16.7	108	109	763	10.6	708
76.17	16.8	110	109.1	778	10.59	707
77.32	16.9	111	109.5	789	10.6	708
86.02	17.2	111	109.6	786	10.57	710
96.00	17.2	111	109.6	786	10.57	710

Table S3: The monitoring parameters for 120% TDG supersaturation group in survival experiment. All the data was recorded at least once per day. The mean TDG saturation was 119.2 ± 0.9%. The mean DO level was 115.8 ± 3.7%. The dashes mean data was not recorded.

Exposure time (h)	Temperature (℃)	TDG (%)	DO (%)	TDG (mmHg)	DO (mg/L)	BP (mmHg)
0.00	14.9	120	107.2	866	10.73	719
11.70	15.4	-	110.1	-	11.02	-
24.00	15.5	120	110.3	861	11.02	716
34.93	15.8	118	115.7	838	11.47	709
36.50	-	118	-	838	-	709
47.62	15.9	119	118.8	849	11.76	711
53.32	16.3	118	117.5	832	11.52	707
62.50	16.3	119	116.7	846	11.42	710
70.88	16.4	120	117.9	852	11.54	709
75.13	16.7	118	118.5	838	11.51	708
76.17	16.8	120	117.9	846	11.44	708
77.32	16.9	120	118.3	849	11.45	708
86.02	17.2	120	118.1	857	11.37	710
96.00	17.2	120	118.1	857	11.37	710

Table S4: The monitoring parameters for 125% TDG supersaturation group in survival experiment. All the data was recorded at least once per day. The mean TDG saturation was 124.4 ± 0.9%. The mean DO level was 119.2 ± 4.6%. The dashes mean data was not recorded.

Exposure time (h)	Temperature (℃)	TDG (%)	DO (%)	TDG (mmHg)	DO (mg/L)	BP (mmHg)
0.00	15	126	110.2	906	11.12	720
11.70	15.4	-	110.5	-	11.06	-
24.00	15.5	123	112.7	881	11.24	716
34.93	15.8	124	121.7	876	12.17	709
36.50	-	125	-	889	-	710
47.62	15.9	125	124	886	12.27	712
53.32	16.3	125	120.2	889	11.76	708
62.50	16.3	123	120.9	874	11.85	710
70.88	16.4	125	122.3	883	11.97	709
75.13	16.7	123	121.3	867	11.79	708
76.17	16.8	124	121	878	11.74	709
77.32	16.9	124	121.5	875	11.77	708
86.02	17.2	125	121.9	886	11.75	710
96.00	17.2	125	121.9	886	11.75	710

Table S5: The monitoring parameters for 130% TDG supersaturation group in survival experiment. All the data was recorded at least once per day. The mean TDG saturation was 129.4 ± 1.1%. The mean DO level was 124.1 ± 3.3%. The dashes mean data was not recorded.

Exposure time (h)	Temperature (℃)	TDG (%)	DO (%)	TDG (mmHg)	DO (mg/L)	BP (mmHg)
0.00	15	131	118	944	11.91	719
11.70	15.4	-	118.8	-	11.88	-
24.00	15.5	131	119	935	11.88	715
34.93	15.8	128	125	909	12.4	709
36.50	-	128	-	912	-	710
47.62	15.9	129	127.1	918	12.57	711
53.32	16.3	129	122.8	912	12.04	709
62.50	16.3	130	126.8	923	12.41	710
70.88	16.4	131	128.2	932	12.54	709
75.13	16.7	129	124.9	915	12.14	709
76.17	16.8	128	125	909	12.13	708
77.32	16.9	128	125.1	909	12.11	708
86.02	17.2	130	126.2	923	12.16	711
96.00	17.2	130	126.2	923	12.16	711

Table S6: The monitoring parameters for 135% TDG supersaturation group in survival experiment. All the data was recorded at least once per day. The mean TDG saturation was 135.2 ± 1.1%. The mean DO level was 129.3 ± 4.6%. The dashes mean data was not recorded.

Exposure time (h)	Temperature (℃)	TDG (%)	DO (%)	TDG (mmHg)	DO (mg/L)	BP (mmHg)
0.00	15	136	120	972	12.1	717
11.70	15.4	-	121.8	-	12.18	-
24.00	15.5	133	121.8	953	12.16	716
34.93	15.8	135	130.8	958	12.97	710
36.50	-	137	-	969	-	710
47.62	15.9	134	133.4	952	13.19	711
53.32	16.3	136	129.9	966	12.71	709
62.50	16.4	134	131.6	952	12.89	712
70.88	16.4	136	131.8	961	12.9	709
75.13	16.7	134	130.8	946	12.72	709
76.17	16.8	135	131.7	957	12.78	708
77.32	16.9	135	131.8	958	12.75	708
86.02	17.2	136	133	966	12.8	709
96.00	17.2	136	133	966	12.8	709

Table S7: The monitoring parameters for 140% TDG supersaturation group in survival experiment. All the data was recorded at least once per day. The mean TDG saturation was 139.7 ± 0.9%. The mean DO level was 133.3 ± 4.4%. The dashes mean data was not recorded.

Exposure time (h)	Temperature (℃)	TDG (%)	DO (%)	TDG (mmHg)	DO (mg/L)	BP (mmHg)
0.00	15	140	125.5	1006	12.66	719
11.70	15.4	-	125.5	-	12.55	-
24.00	15.5	140	125.6	1004	12.53	716
34.93	15.8	141	136.4	998	13.52	709
36.50	-	140	-	992	-	710
47.62	15.9	140	138.3	992	13.66	711
53.32	16.3	138	135.5	978	13.28	707
62.50	16.4	140	135.1	992	13.24	710
70.88	16.4	141	136.3	998	13.34	709
75.13	16.7	139	134.3	981	13.06	709
76.17	16.8	139	134.9	984	13.09	708
77.32	16.9	138	133.9	978	12.96	708
86.02	17.2	140	135.7	992	13.06	710
96.00	17.2	140	135.7	992	13.06	710

Table S8: The number of experimental fish in 125%, 130%, 135% and 140% TDG groups in survival experiment and the body length and mass of dead fish. The fish number was 40 in each TDG group of 100%, 110% and 120% while the body parameters were not measured due to no mortality. The data of body length and mass is shown as mean ± SE.

	140%	135%	130%	125%
Total number	40	31	40	31
Body length (mm)	47.2±1.2	45.7±1.0	46.3±1.3	45.0
Mass (g)	2.33±0.13	2.05±0.14	2.20±0.18	1.90

Table S9: The monitoring parameters for 100% TDG group in swimming ability experiment. All data was recorded at least once per day. The mean TDG saturation was 101.6 ± 0.9%, and the mean DO level was 94.8 ± 2.5%. The dashes mean data was not recorded.

Exposure time (h)	Temperature (℃)	TDG (%)	DO (%)	TDG (mmHg)	DO (mg/L)	BP (mmHg)
0.00	16.2	101	90.7	716	9.13	710
3.17	16.4	100	94.8	722	9.43	719
22.48	17.7	102	98.7	728	9.65	713
28.90	17.8	103	95.2	722	9.24	702
46.42	17.2	102	94.4	721	9.30	709

Table S10: The monitoring parameters for 110% TDG supersaturation group in swimming ability experiment. All data was recorded at least once per day. The mean TDG saturation was 110.9 ± 2.0%, and the mean DO level was 106.7 ± 2.7%. The dashes mean data was not recorded.

Exposure time (h)	Temperature (℃)	TDG (%)	DO (%)	TDG (mmHg)	DO (mg/L)	BP (mmHg)
0.00	16.6	111	107.3	910	9.86	819
2.37	14.6	-	113.1	-	10.38	-
6.97	15.3	111	107.7	909	9.84	818
22.27	14.7	112	107.5	916	9.85	820
24.85	14.7	-	103.9	-	9.52	-
34.18	15.1	108	-	885	-	822
35.35	15.7	108	-	766	-	708
47.53	15.7	110	106.0	780	9.6	707
55.40	16.6	109	-	769	-	704
72.60	15.9	113	105.0	800	9.45	705
80.13	16	112	110.3	786	9.84	702
95.42	16.3	114	-	798	-	702
101.88	16.6	108	-	758	-	699
105.52	16.4	112	-	783	-	702
119.82	16.6	114	102.6	808	9.12	707
127.43	16.9	112	105.4	790	9.29	706
148.25	16.6		106.6		9.42	
166.05	16.6	110	105.4	780	9.33	707

Table S11: The monitoring parameters for 120% TDG supersaturation group in swimming ability experiment. All the data was recorded at least once per day. The mean TDG saturation was 119.9 ± 0.8%, and the mean DO level was 115.0 ± 3.5%. The dashes mean data was not recorded.

Exposure time (h)	Temperature (℃)	TDG (%)	DO (%)	TDG (mmHg)	DO (mg/L)	BP (mmHg)
0.00	16.7	120	117.4	984	10.78	819
2.37	14.7	-	122.2	-	11.2	-
6.97	15.3	121	118.2	993	10.79	819
22.27	14.7	119	117.8	979	10.78	819
24.85	14.7	-	113.7	-	10.41	-
34.18	15.8	119	-	843	-	707
35.35	15.7	120	-	849	-	708
47.53	15.7	120	114.0	846	10.33	707
55.40	16.2	119	-	835	-	704
72.60	16	121	111.3	855	10.01	706
80.13	16	119	118.0	832	10.53	701
95.42	16.1	120	-	843	-	702
101.88	16.6	119	-	832	-	700
105.52	16.6	121	-	846	-	700
119.82	16.6	121	109.4	858	9.71	707
127.43	16.9	120	113.1	844	9.97	705
148.25	16.7		112.9		9.97	
166.05	16.6	120	112.2	846	9.93	707

Table S12: The monitoring parameters for 125% TDG supersaturation group in swimming ability experiment. All the data was recorded at least once per day. The mean TDG saturation was 124.3 ± 0.9%, and the mean DO level was 118.7 ± 3.8%. The dashes mean data was not recorded.

Exposure time (h)	Temperature (℃)	TDG (%)	DO (%)	TDG (mmHg)	DO (mg/L)	BP (mmHg)
0.00	16.7	126	122.8	1030	11.28	819
2.37	14.7	-	126.1	-	11.56	-
6.97	15.4	125	122.2	1016	11.16	818
22.27	14.7	122	121.7	1004	11.14	820
24.85	14.7	-	117.5	-	10.75	-
34.18	15.8	124	-	875	-	706
35.35	15.8	125	-	883	-	708
47.53	15.7	124	117.4	878	10.62	707
55.40	16.1	124	-	872	-	705
72.60	16	124	115.9	878	10.42	706
80.13	16	124	120.9	869	10.78	701
95.42	16.1	124	-	872	-	702
101.88	16.7	125	-	872	-	699
105.52	17	124	-	872	-	701
119.82	16.6	124	112.2	878	9.96	707
127.43	16.9	125	115.3	882	10.16	705
148.25	16.7		117.1		10.33	
166.05	16.6	124	115.5	878	10.22	70

Table S13: The monitoring parameters for 130% TDG supersaturation group in swimming ability experiment. All the data was recorded at least once per day. The mean TDG saturation was 130.9 ± 1.2%, and the mean DO level was 122.8 ± 2.8%. The dashes mean data was not recorded.

Exposure time (h)	Temperature (℃)	TDG (%)	DO (%)	TDG (mmHg)	DO (mg/L)	BP (mmHg)
0.78	17.4	128	128.4	908	12.86	707
8.92	17.5	-	120.1	-	12.01	-
26.32	-	131	-	925	-	708
36.95	18.2	131	134.4	932	13.24	710
55.78	-	130.8	-	-	-	-
62.33	18.3	132	127.0	930	12.46	707
75.67	-	131	-	927	-	707
81.52	18.9	-	119.8	-	11.62	-
84.57	19	132	122.2	936	11.85	709
96.00	19.3	131	126.5	933	12.18	713

Table S14: The monitoring parameters for 135% TDG supersaturation group in swimming ability experiment. All the data was recorded at least once per day. The mean TDG saturation was 134.8 ± 0.4%, and the mean DO level was 129.3 ± 3.7%. The dashes mean data was not recorded.

Exposure time (h)	Temperature (℃)	TDG (%)	DO (%)	TDG (mmHg)	DO (mg/L)	BP (mmHg)
0.00	-	134	-	944	-	706
8.92	19.4	135	128.3	967	12.78	714
17.03	19.7	135	132.5	959	13.11	710
35.53	20.5	135	131.4	961	12.80	712
42.23	-	135	-	959	-	711
57.10	21.1	135	124.0	961	11.94	710

Table S15: The monitoring parameters for 140% TDG supersaturation group in swimming ability experiment. All the data was recorded at least once per day. The mean TDG saturation was 140.3 ± 0.5%, and the mean DO level was 133.9 ± 2.9%. The dashes mean data was not recorded.

Exposure time (h)	Temperature (℃)	TDG (%)	DO (%)	TDG (mmHg)	DO (mg/L)	BP (mmHg)
0.52	16.9	141	137.0	1004	13.56	714
17.08	17.5	140	131.9	993	12.89	709
28.90	17.4	-	136.5	-	13.37	-
36.90	17.5	140	130.2	992	12.73	709

Table S16: The monitoring parameters for recovery group in swimming ability experiment. The mean TDG saturation was 101.5 ± 0.5%, and the mean DO level was 94.4 ± 1.5%.

	Recovery time (h)	Temperature (℃)	TDG (%)	DO (%)	TDG (mmHg)	DO (mg/L)	BP (mmHg)
130%	0	21.2	101	93.7	720	8.14	710
	22.83	22.5	102	95.7	723	8.22	707
135%	0.00	20.5	102	98.0	727	8.71	713
	8.63	21.8	101	93.4	720	8.11	713
	45.45	18.9	101	93.5	720	8.60	712
140%	0	17.5	102	94.4	721	8.6	708
	17.40	17.7	102	93.2	719	8.59	708
	28.03	18.2	101	93.5	720	8.55	714

Table S17: The number of experimental fish used to test U_{crit} in each group under non-lethal TDG supersaturation exposure and the corresponding body length and mass. 0 d refers to the control group (100% TDG). The data of body length and mass is shown as mean ± SE.

		0 d	1 d	2 d	3 d	4 d	5 d	6 d	7 d
110%	Number	7	7	7	7	7	7	7	7
	Body length (mm)	55.4±1.4	56.9±3.4	61.0±2.5	56.1±1.8	57.7±2.1	58.1±2.8	59.1±2.6	58.0±1.7
	Mass (g)	3.89±0.29	4.21±0.77	4.17±0.53	4.13±0.39	4.64±0.51	4.71±0.78	4.80±0.60	4.07±0.32
120%	Number	7	7	7	7	7	7	7	7
	Body length (mm)	55.4±1.4	58.7±2.3	58.7±1.2	56.1±1.1	59.7±2.2	59.0±1.3	56.1±2.2	58.9±2.7
	Mass (g)	3.89±0.29	4.47±0.59	3.80±0.26	3.73±0.27	5.21±0.47	4.10±0.34	3.66±0.60	4.53±0.59
125%	Number	7	7	7	7	7	7	7	7
	Body length (mm)	55.4±1.4	58.7±1.2	57.3±2.5	59.6±1.9	58.6±2.0	60.7±1.1	57.4±1.8	55.6±1.4
	Mass (g)	3.89±0.29	4.61±0.45	3.91±0.27	4.74±0.49	4.91±0.50	4.49±0.32	4.77±0.61	3.93±0.34

Table S18: The number of experimental fish used to test U_{crit} in each group under lethal TDG supersaturation exposure and the corresponding body length and mass. 0 h refers to the control group (100% TDG). The data of body length and mass is shown as mean ± SE. The dashes mean no data at corresponding conditions.

140%	Exposure time	0 h	0.8 h	1.6 h	3.2 h	6.3 h	10.4 h	17.8 h		
	Number	7	6	5	5	6	6	5		
	Body length (mm)	55.4±1.4	55.0±2.0	49.8±3.8	54.6±5.3	59.0±4.0	48.8±3.1	64.0±2.8		
	Mass (g)	3.89±0.29	3.29±0.37	2.49±0.59	3.57±0.78	4.06±0.80	2.51±0.44	5.43±0.64		
135%	Exposure time	0 h	7.4 h	14.8 h	29.3 h	42.9 h	50.8 h	56.5 h		
	Number	7	6	6	7	6	6	6		
	Body length (mm)	55.4±1.4	55.8±2.3	57.7±3.4	57.1±3.8	52.2±3.6	55.0±3.3	60.2±3.6		
	Mass (g)	3.89±0.29	3.18±0.30	3.62±0.45	3.93±0.64	2.86±0.46	3.42±0.40	3.67±0.66		
130%	Exposure time	0 h	5.8 h	11.6 h	23.3 h	52.0 h	60.7 h	72.5 h	80.8 h	95.9 h
	Number	7	6	5	6	6	7	5	6	6
	Body length (mm)	55.4±1.4	53.8±2.7	53.0±3.7	56.8±2.2	54.7±3.4	55.3±3.4	55.0±3.2	59.8±1.9	55.0±2.2
	Mass (g)	3.89±0.29	3.08±0.51	2.82±0.63	3.62±0.40	2.92±0.51	2.82±0.52	2.72±0.40	3.29±0.36	2.66±0.32

Table S19: The number of experimental fish used to test U_{crit} in each recovery group and the corresponding body length and mass. 0 h refers to the control group (100% TDG). The data of body length and mass is shown as mean ± SE. The dashes mean no data at corresponding conditions.

		0 h	24 h	48 h
140%	Number	7	4	3
	Body length (mm)	55.4 ± 1.4	57.8 ± 3.2	56.7 ± 5.0
	Mass (g)	3.89 ± 0.29	3.95 ± 0.61	3.16 ± 0.90
135%	Number	7	7	7
	Body length (mm)	55.4 ± 1.4	54.9 ± 2.6	55.6 ± 2.7
	Mass (g)	3.89 ± 0.29	3.14 ± 0.49	3.28 ± 0.35
130%	Number	7	6	-
	Body length (mm)	55.4 ± 1.4	56.0 ± 2.8	-
	Mass (g)	3.89 ± 0.29	3.33 ± 0.52	-

Table S20: Survival analysis of bighead carp under different TDG supersaturation. The significance value is set as $p < 0.05$, and the * followed the *p* value indicated that there was a significant difference.

	100%		110%		120%		125%		130%		135%		140%	
	χ2	*p*	*χ2*	*p*	*χ2*	*p*	*χ2*	*p*	*χ2*	*p*	*χ2*	*p*	*χ2*	*p*
100%							1.290	0.256	14.013	0.000*	35.858	0.000*	69.134	0.000*
110%							1.290	0.256	14.013	0.000*	35.858	0.000*	69.134	0.000*
120%							1.290	0.256	14.013	0.000*	35.858	0.000*	69.134	0.000*
125%	1.290	0.256	1.290	0.256	1.290	0.256			7.940	0.005*	23.681	0.000*	49.958	0.000*
130%	14.013	0.000*	14.013	0.000*	14.013	0.000*	7.940	0.005*			7.353	0.007*	35.239	0.000*
135%	35.858	0.000*	35.858	0.000*	35.858	0.000*	23.681	0.000*	7.353	0.007*			10.492	0.001*
140%	69.134	0.000*	69.134	0.000*	69.134	0.000*	49.958	0.000*	35.239	0.000*	10.492	0.001*		
